# Effect of Slurry Thickness on the Quality of Aluminized Coatings

**DOI:** 10.3390/ma15196758

**Published:** 2022-09-29

**Authors:** Zhuoyue Li, Cheng Wang, Xiangyu Ding, Xin Li, Jiabo Yu, Qiuliang Li, Yi Qu

**Affiliations:** 1 Fundamental Department, Air Force Engineering University, Xi’an 710051, China; 2 College of Aircraft Engineering, Nanchang Hangkong University, Nanchang 330063, China

**Keywords:** aluminized coating, coating thickness, diffusion, roughness, slurry thickness

## Abstract

Diffusion aluminum coating is crucial to protect aero-engine turbine blades from high-temperature oxidation. Slurry aluminizing, as a commonly-used coating preparation technology, has variations in the process parameters that directly affect the quality of the coating. Therefore, this paper investigates the effect of slurry thickness on coating quality. Different forms of aluminized coatings were obtained by coating nine DZ22B nickel-based superalloy plates of the same size with different slurry thicknesses while keeping other parameters constant. These aluminized coatings were characterized using a scanning electron microscope (SEM) with an energy dispersive spectrometer (EDS), an X-ray diffractometer (XRD), and a surface gauge. The results show that the AlNi phase dominates the matrix of the aluminized coating, and the outer layer of the coating has white dotted precipitates of Cr. As the slurry thickness increases, the coating thickness increases, and the proportion of the outer layer in the overall coating increases. In contrast, the thickness of the interdiffusion layer does not change significantly. The thicker the slurry, the higher the Al content of the coating surface. A medium-thickness slurry can form a smooth aluminizing coating with a roughness Ra < 4.5 μm surface. The combined results show that a medium-thick slurry can produce a high-quality coating.

## 1. Introduction

The core parameter of an aircraft engine thrust increase is the pre-turbine gas temperature. The limiting factor for increasing pre-turbine gas temperatures is the material’s high-temperature resistance (1300–2000 K) [1]. Nickel-based superalloys have excellent mechanical properties at high temperatures (900–1600 K), which is why they are most widely accepted in manufacturing engine turbine blades [2,3]. The adjustment of alloy composition can improve the alloy properties to some extent. For example, adding Cr, Al, and Y can confer oxidation resistance; B, Zr, C, and H can refine grain boundaries; B and Ta can improve creep properties; and Co, Cr, and Si can provide anti-sulfuration properties [4]. The high-temperature oxidation resistance of superalloys is positively correlated with the content of the corresponding alloying elements, but simply increasing the content of antioxidant elements can significantly reduce the mechanical properties of the alloy. The coating’s application balances the material’s mechanical properties and high-temperature oxidation resistance [1,5,6,7]. Aluminide coatings or modified aluminide coatings (AlSi [8], PtAl [9], AlSiY [10]) are commonly used to provide high-temperature oxidation resistance to the blades.

The relatively mature methods for preparing aluminide coatings include thermal spraying [11], pack cementation [12,13], slurry cementation [14], and hot-dipping [8]. The slurry method has several advantages over other methods: 1. shorter thermal cycle time for preparing the coating; 2. the possibility of localized coating, for example, avoiding the blade root of the turbine blade to coat the blade body; and 3. the possibility of coating large parts [15]. Therefore, the slurry method is commonly used to obtain diffusion aluminide coatings for turbine blades and fans [16,17,18]. The slurry is a suspension formed by mixing the aluminum powder with a binder. The slurry is sprayed on the substrate, dried and cured, and then heat-treated to obtain a diffused aluminum coating [19]. Therefore, parameters such as slurry composition, deposition thickness, temperature, and heat treatment time can influence the organization and properties of diffused aluminum coatings. Montero et al. [20] investigated the effects of exposure atmosphere, substrate composition, and surface treatment on aluminum coatings on steel surfaces. The results showed that the Ar atmosphere protects against oxidation to obtain intact coatings, that an increase in Cr content reduces coating coverage, and that higher roughness and smaller particle size facilitate the formation of thick and uniform aluminum coatings. Rasmussen et al. [21] investigated the microstructure of aluminide coatings formed by coating the surface of nickel-based high-temperature alloys with two different thicknesses of slurry. The results show that slurry thickness significantly affects the morphology and composition of the coatings. The mechanism of aluminide coating formation on pure nickel or nickel-based alloy surfaces is well understood [22], but the optimization of slurry aluminizing process parameters is under continuous research [13,19,20].

In the present paper, nine specimens were sprayed with different thicknesses of slurry to investigate the effect of slurry thickness on the quality of aluminized coatings. The variation of coating quality with slurry thickness was obtained by microstructure observation, coating thickness measurement, physical phase analysis, and coating surface roughness inspection. The investigation results can be used as a reference for the selection of slurry aluminizing process parameters.

## 2. Materials and Methods

**Figure 1:**
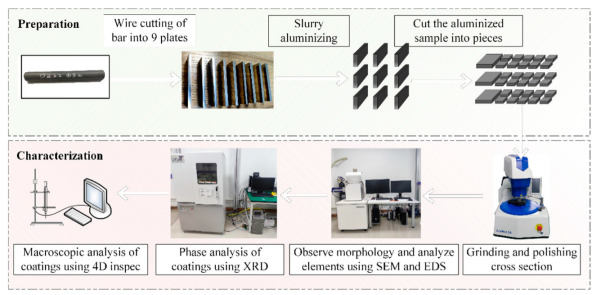
Flow chart of specimen preparation and characterization.

We carried out different thicknesses of slurry aluminizing on the surface of DZ22B nickel-based, high-temperature alloy to study the effect of slurry thickness on the coating’s thickness, morphology, and composition. The experimental flow is divided into preparation and characterization, as shown in Figure 1.

### 2.1. Aluminized Specimens Preparation

The DZ22B alloy with good high-temperature mechanical properties and stability was chosen as the substrate, and its nominal composition and EDS analysis composition are shown in Table 1 [23]. The DZ22B alloy bar with a diameter of 32 mm was cut into 9 flat pieces of the same size (80 mm× 20 mm × 5 mm) using a wire cutting machine (DK7725H, ZHCF, Suzhou, China). The flat surfaces were abraded using a grinding and polishing machine (EcoMet 30, Buehler, Lake Bluff, IL, USA). The speed of the chassis was set at 300 rpm, the sandpapers P240/P800 were used, and the grinding and polishing time was determined by the polishing results. Due to the size limitation of the flat specimens, it was impossible to mount the specimens in the fixture, so manual grinding and polishing was used. The surface roughness to meet the aluminizing conditions is 0.044 μm [24].

**Figure 2:**
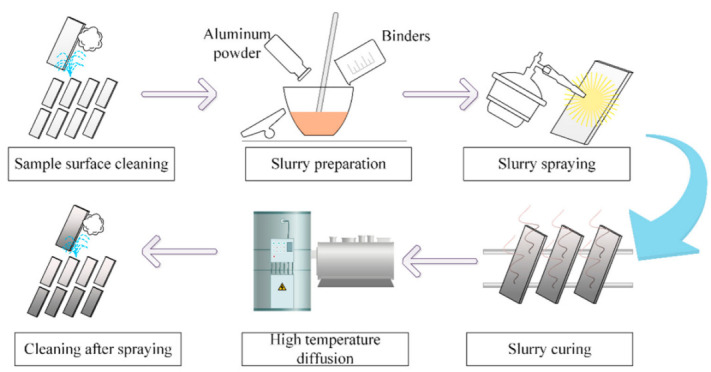
Slurry aluminizing process.

The slurry aluminizing procedure is shown in Figure 2. After the plate specimens were ground and polished smooth, they were cleaned in acetone using an ultrasonic cleaner (KM-410C, KJM, Guangzhou, China) for 5 min, dried, and set aside. Pure aluminum powder was used as the permeate, and fully ground in a mortar and pestle for two hours. The binder consisted of isoamyl acetate, diethyl oxalate, and nitrocellulose. The ground permeate and binder were mixed to form a slurry. The next step was slurry spraying, and nine specimens were sprayed with different thicknesses of slurry, as shown in Table 2. After the slurry curing, we conducted two stages of high-temperature diffusion treatment in an argon atmosphere on the specimens: I. 950 ± 10 °C, 1 h 10 min; II. 1000 ± 10 °C, 2 h 20 min. This parameter is consistent with the typical surface aluminizing of turbine blades. Finally, the aluminized specimens were cleaned to remove surface impurities and residual slurry. After obtaining the aluminized specimens, each specimen was cut into 32 slices with the same dimensions (10 mm × 5 mm × 4 mm) for subsequent characterization experiments.

### 2.2. Aluminized Coating Characterization

The slices obtained by the preparation process were mounted, and the cross-sections were polished using an automatic grinding and polishing machine (EcoMet30, Buehler, Lake Bluff, IL, USA). After taking out the slices from the mounts, they were cleaned and dried in an ultrasonic cleaner (KM-410C, KJM, Guangzhou, China). The thickness and morphology of the coating on the cross-section of the slices were observed using an SEM (VEGA-3 XMU, TESCAN, Brno, Czech Republic). The parameters of the SEM were set as follows: accelerating voltage 20 kV, current 3 nA, working distance 20 mm, Image resolution 768 × 665 px, magnification 500×, SE detector for morphology observation, and BSE detector for coating thickness measurement. Elemental composition analysis was performed using the EDS accompanying the SEM. Each slice gives the coating thickness on both sides so that each specimen can get 64 coating thickness values, and 9 specimens can get 576 sets of data in total. The coating thickness values were measured using Image J software developed by the National Institutes of Health. These data were used to determine the relationship between slurry thickness and coating thickness. A physical phase analysis of the coatings was performed using an XRD (XRD6100, SHIMADZU, Kyoto, Japan) with parameters of Cu Kα radiation, λ = 0.15418 nm, 2θ = 20°–80°. The coating surface was analyzed using a surface gauge (4D InSpec, 4D Technology, Tucson, AZ, USA) to investigate the effect of slurry thickness on coating roughness.

## 3. Results and Discussion

### 3.1. Thickness of Aluminized Coating

**Figure 3:**
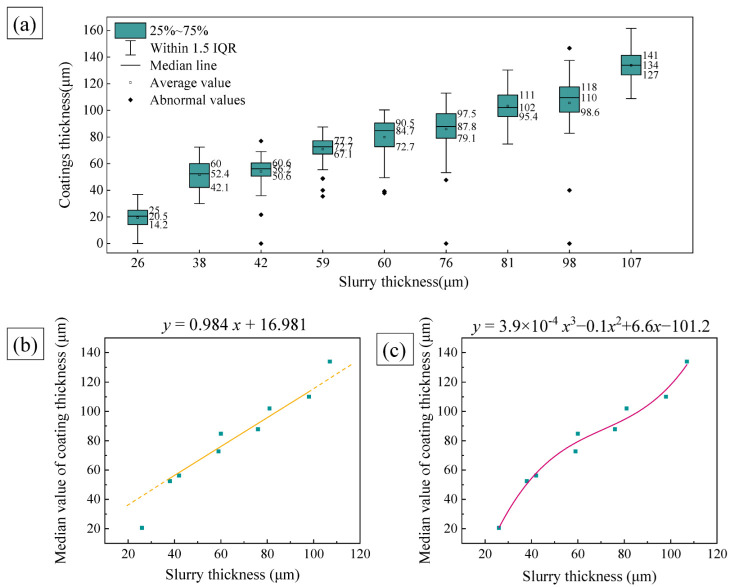
Relationship between slurry thickness and coating thickness. (**a**) Box plot of slurry thickness vs. coating thickness; (**b**) linear fitting curve of slurry thickness and coating thickness; and (**c**) cubic polynomial fitting curve of slurry thickness and coating thickness.

Each specimen was cut into 32 slices and 64 coating thickness values were obtained. The slurry thickness applied to each specimen was plotted against its corresponding coating thickness in a box plot, as shown in Figure 3. The box plot was plotted using OriginPro software (2018C, OriginLab Corporation, Northampton, MA, USA). According to the definition of the box plot, the lower boundary of the box is the lower quartile (Q_1_), the horizontal line inside the box is the median of the sample (Q_2_), and the upper boundary of the box is the upper quartile (Q_3_). The interquartile distance (IQR) is equal to (Q_3_ − Q_1_). The upper edge of the whisker line is the upper limit of the normal value, and its value is (Q_3_ + 1.5IQR). The lower edge of the whisker line is the lower limit of the normal value, and its value is (Q_1_ − 1.5IQR). In this paper, coating thickness values within the interval of [Q_1_ − 1.5IQR, Q_3_ + 1.5IQR] are considered normal values. Otherwise, they are considered abnormal values. From Figure 3a, it can be found that there are abnormal coating thickness values for specimens #3–6 and specimen #8. Those cases of thin or even no coating thickness in abnormal values are probably because the areas are located in the clamping position of the specimen during aluminizing and are not sprayed with slurry or the sprayed slurry is thin. Those thicker coatings’ abnormal values are caused by the aggregation of the slurry at the edges during the curing process.

The median coating thickness was slightly above the mean value except for specimen #7, where the abnormal values influenced the mean value. Therefore, the median line can more fairly reflect the interval where the coating thickness values are most concentrated. We made a linear fit curve and a cubic polynomial fit of the median slurry thickness versus coating thickness in Figure 3b,c, respectively. The goodness-of-fit (*R*^2^) of the linear fit was 0.93892, while the *R*^2^ of the cubic polynomial fit was 0.98346. Furthermore, in Figure 3b, we can see that the coating thickness values for specimen #1 and specimen #9 deviate far from the linear fit line. Y. Tamarin [15] proposed a 1:1 relationship between slurry thickness and coating thickness after diffusion annealing of the cured slurry coating at a high temperature (1200 °C) for 1–2 h. From our experimental results, this linear relationship can be applied when the slurry coating is of medium thickness. However, it does not apply when the slurry thickness is too thin or too thick, which may be related to the diffusion dynamics. When the slurry layer is thicker, the diffusion dynamics are strong, and the interdiffusion between Ni and Al is fast, forming a thicker coating, while the opposite is true when the slurry thickness is thin.

### 3.2. Microstructure and Element Distribution

**Figure 4:**
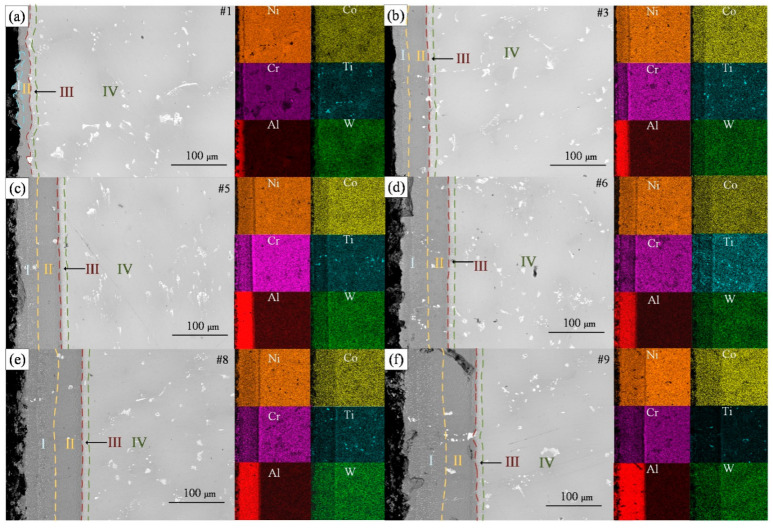
BSE cross-sectional images of different thicknesses of aluminized coatings on the surface of DZ22B alloy. (**a**) 23 ± 9 μm, (**b**) 60 ± 5 μm, (**c**) 85 ± 5 μm, (**d**) 89 ± 6 μm, (**e**) 109 ± 12 μm, (**f**) 136 ± 13 μm.

Typical slices from nine specimens were selected to observe the microstructure of their cross-sections. The coating thickness and the distribution of elements on the cross-sections were obtained, as shown in Figure 4. For brevity, only the BSE images of the six samples #1, #3, #5, #6, #8, and #9 and the distribution of the major elements are shown in Figure 4.

**Figure 5:**
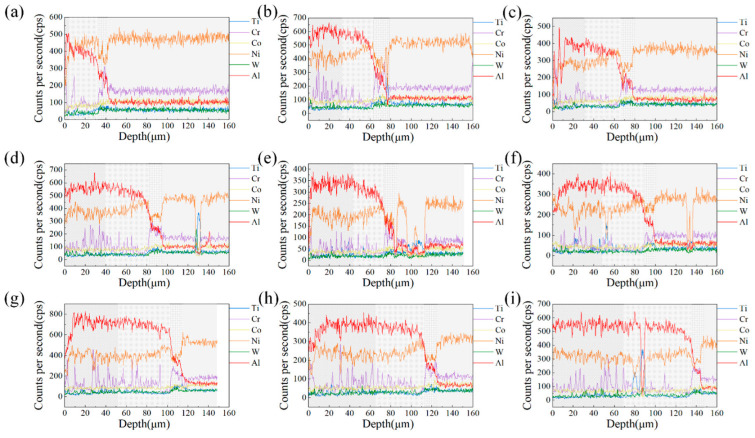
The elements’ content variation along the coating depth direction. (**a**–**i**) represent specimens #1–9, respectively.

For specimen #1, the coating formed at a slurry thickness of only 26 μm was discontinuous, showing high and low undulations. As the slurry thickness increases, the boundary between the coating and the substrate becomes flat and straight, and the coating becomes thick. The top of the thicker aluminized layer is porous [25]. Overall, the slice cross-section can be divided into four regions, as shown by the markers in Figure 4. Zone I shows the same dark gray color as zone II under the BSE detector. However, there are dense white spots in zone I, which are produced by Cr elements precipitated from the substrate forming Cr*_x_*Al*_y_* compounds with Al [22]. In Figure 5, the elemental content of Cr fluctuates significantly from high to low in zone I, decreases in amplitude in zone II, increases abruptly to a higher level in zone III, and plateaus in zone IV. It further proves that the small white dots in zone I are Cr compounds. The Ni element shows a slow increase in zone I and II, while the Al element, on the contrary, is slowly decreasing. Therefore, the matrix in zone I may be an Al-rich AlNi phase, while zone II is a Ni-rich AlNi phase [21], which needs to be verified after XRD testing. Zone III is the zone of the sharp decline of Al elements and the enrichment of alloying elements in the substrate. It is noteworthy that the content of Ni in zone III is lower than its content in the other zones. Zone III is often referred to as the interdiffusion zone (IDZ) [26], while zone IV is the DZ22B alloy substrate. Based on the EDS map attached next to the BSE image of each sample, it is clear that the larger white spots shown on the substrate and coating are compounds of Ti.

Figure 4a shows the coating formed by the thin slurry, with zone I only sporadically distributed at the edges. As the thickness of the slurry increases, zone I takes up an increasing proportion of the coating system and gradually overtakes zone II. It is probably due to the incomplete diffusion of Ni in the slurry with increasing slurry thickness that leads to a slight increase of Al content in the coating. At the same time, Cr solubility in the AlNi phase decreases with increasing Al content [27], forming more white dot precipitates. As shown in Figure 4, the thickness of the IDZ layer does not vary with the slurry thickness and always remains around 15 μm. This is because the thickness of the IDZ layer is mainly affected by the temperature and time of high-temperature diffusion [28]. The same heat treatment method was used for all specimens in this work and therefore did not affect the IDZ layer.

### 3.3. Physical Phase Analysis

**Figure 6:**
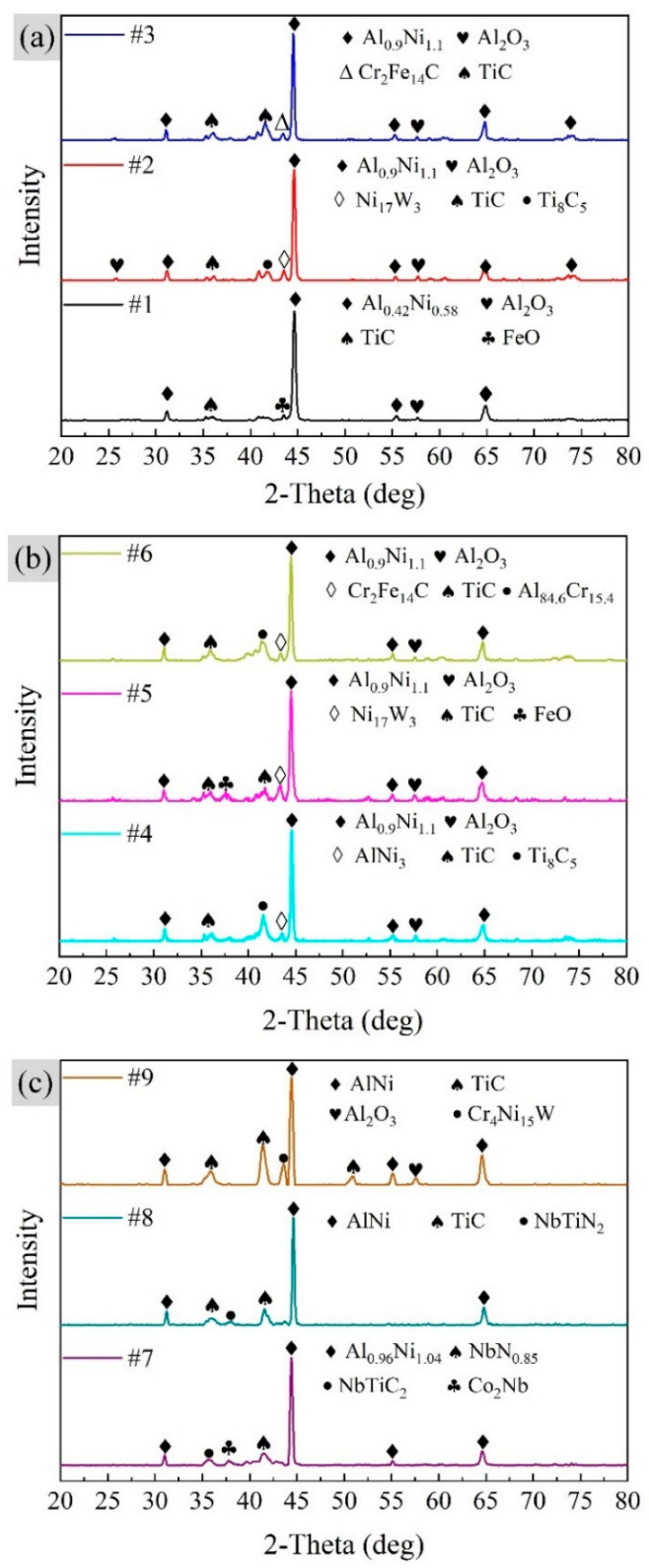
XRD patterns for aluminized specimens. (**a**) #1–3 specimens, (**b**) #4–6 specimens, and (**c**) #7–9 specimens.

**Figure 7:**
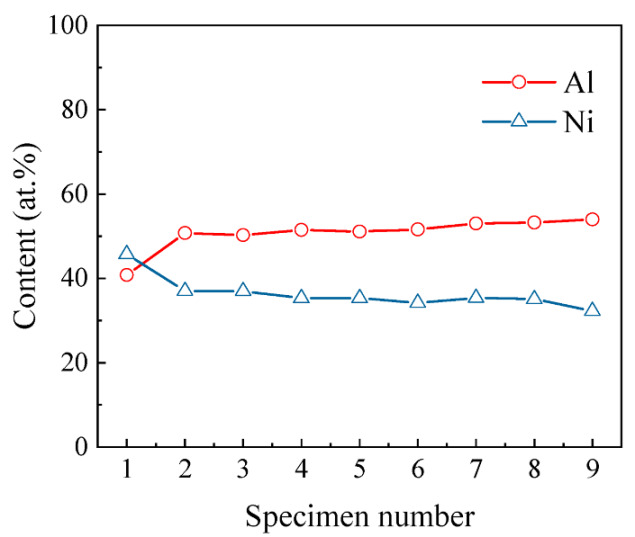
The variation pattern of Al and Ni elements in the aluminized specimens’ outermost layer (zone I).

Slurry aluminizing coatings are usually divided into high-temperature (850–1100 °C) low-activity and low-temperature (700–850 °C) high-activity coatings [15]. The former is dominated by the AlNi phase [29], while the latter is dominated by the Al_3_Ni, Al_3_Ni_2_, and AlNi phase from the surface to the interior [30]. Figure 6 shows the XRD patterns for the surfaces of nine slurry aluminized specimens. The main peak of each specimen is the AlNi compound, which is due to the high-temperature low-activity coating generated by the heat treatment of all specimens at temperatures greater than 950 °C. However, there is a slight change in the molecular formula of the AlNi compound as the slurry thickness increases. From specimen #1 to specimen #9, the molecular formula changes as follows: Al_0.42_Ni_0.58_(#1) → Al_0.9_Ni_1.1_(#2–#6) → Al_0.96_Ni_1.04_(#7) → AlNi(#8, #9). The Ni-rich AlNi phase dominates the coating matrix when the slurry thickness is thin. With the increase of the slurry thickness, the Al element content of the outermost layer of the coating gradually increased, the Ni element content gradually decreased (Figure 7), and the AlNi phase of the matrix was no longer Ni-rich, and a 1:1 molecular formula was obtained. In Figure 7, the atomic percentages of Al are higher than those of Ni except for specimen #1, which may be due to the formation of compounds of Al with other elements. For example, the Al_2_O_3_ phase marked in Figure 6 is generated by the oxidation of Al in the AlNi phase. This oxidation reaction produces the Al_2_O_3_ film, which is the essence of the oxidation resistance of the aluminized coating. In addition, the Al_84.6_Cr_15.4_ phase was marked in specimen #6, which is consistent with the statement that AlCr compounds were formed in zone I, as mentioned in the previous section. For the TiC and Ti_8_C_5_ phases, it corresponds to the large white spots in Figure 4.

### 3.4. Surface Roughness

**Figure 8:**
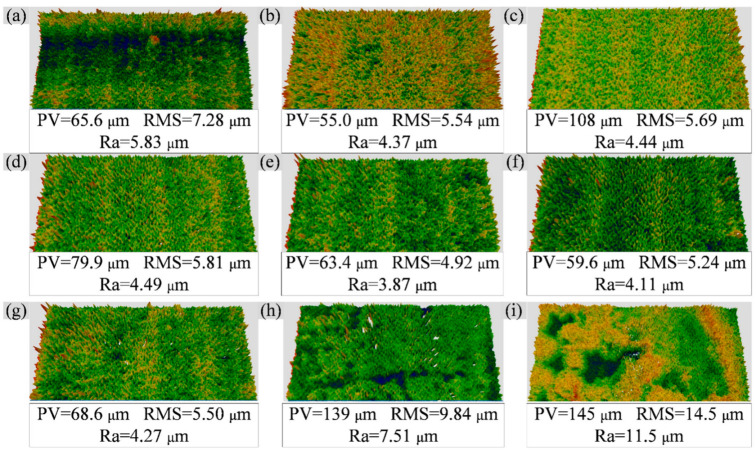
Surface macromorphology and roughness of aluminized specimens. (**a**–**i**) are specimens #1–9 in order.

Figure 8 shows the surface morphology [31] and roughness values of the aluminized specimens. In Figure 8, PV indicates the difference between peak and valley values, and RMS and Ra are roughness parameters. In addition, there are surface stats parameters such as skewness (Ssk), kurtosis (Sku), maximum valley depth (Sv), and the maximum height of the surface (Sq) in Table 3. We assume that the roughness of the DZ22B specimens before aluminizing is the same due to using the same sanding method (P800sandpaper). However, in Figure 8, the coating surface morphology and roughness changed considerably with the change in slurry thickness. As the slurry’s thickness increases, the coating surface’s roughness first decreases and then increases. From the three-dimensional morphology, the unevenness of specimens #1–7 is caused by tiny needle-like bumps, while the coating surfaces of specimens #8 and #9 are islands or patches of bumps or pits.

The relatively rough macroscopic surface of specimen #1’s aluminized coating corresponds to the undulating coating in Figure 4a. We have two guesses as to the cause of this rough surface. One possibility is related to the particle size of the Al powder. Although the diffusion heat treatment temperature has exceeded the melting point of Al, the Al powder particles may still slightly retain their paste shape. This shape causes undulations at the interface between the coating and the substrate, thus affecting the surface roughness. Another possibility is the overproliferation of Ni. Due to the thin thickness of the slurry, after the complete diffusion of Al and Ni, the Ni in some positions continues to diffuse outward, forming a Ni-rich NiAl phase and an uneven surface. The medium slurry thickness formed a relatively smooth surface (Ra < 4.5 μm), which is consistent with the findings of the literature [32] that found that the low-aluminum activity coating formed a coarse crystalline smooth surface. For the thicker slurry (samples #8 and #9), corresponding to Figure 4e,f, it can be seen that the interface between the coating and the substrate is relatively flat. The oxidation of particles at the top of the slurry, the lower temperature at the top of the coating due to argon flow, and the incomplete diffusion of nickel all contribute to the coating’s loose and porous outer layer [25].

## 4. Conclusions

**Figure 9:**
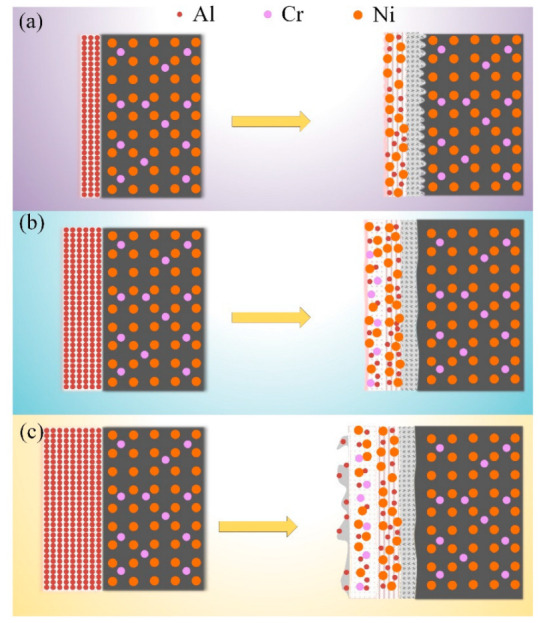
Schematic diagram of slurry aluminization for (**a**) thin, (**b**) medium, and (**c**) thick slurry thickness.

To investigate the effect of slurry thickness on the coating, we analyzed the coating thickness, microstructure, element distribution, physical phase, and surface roughness formed by different slurry thicknesses. Figure 9 shows a schematic diagram of three different thicknesses of slurry aluminized in thin, medium, and thick.

Combining Figure 9 and the experimental results, we obtain the following conclusions.

1. The coating thickness increases with increasing slurry thickness. The relationship between coating and slurry thickness is approximately linear in the medium thickness interval, but not when it is too thick or too thin, which may be related to the diffusion dynamics.
2. The aluminized coating can be divided into an outer layer containing Cr precipitates (Zone I), an inner layer of AlNi phase (Zone II), and an IDZ layer (Zone III). When the slurry is thin, zone I can hardly be observed, and with the increase of slurry thickness, the thickness of zone I gradually increases and exceeds that of zone II. The thickness of zone III does not change with the slurry thickness. When the slurry is relatively thick, a porous aluminized layer will be formed on the outer surface, which will affect the surface roughness of the coating.
3. As the thickness of the slurry increases, the Al content in the coating gradually increases, and the matrix of the aluminized coating gradually becomes an AlNi phase of molecular formula 1:1 from the Ni-rich AlNi phase.
4. The coating surface formed by medium-thickness slurry is smoother than that of thinner or thicker slurry.

Summing up the above conclusions, we found that aluminizing treatment using a medium-thick slurry can obtain coatings with high Al reserves (good oxidation resistance), stable AlNi phases, and smooth surfaces. As for the other performance parameters of the coating, such as fatigue, creep, and experimental verification of oxidation properties, further studies are needed.

## Figures and Tables

**Table 1:** Mass fraction of main elements of DZ22B alloy (wt.%).

Element	Cr	Co	W	Al	Ti	Fe	Nb	Hf	Ni
Nominal	8.0–10.0	9.0–11.0	11.5–12.5	4.75–5.25	1.75–2.25	<0.25	0.75–1.25	0.8–1.1	Bal
EDS	9.25–9.95	9.03–9.97	11.1–12.9	5.41–5.86	1.95–2.40	0.26–0.37	0.95–1.13	0.74–1.77	56.4–58.9

**Table 2:** Slurry thickness values on different specimens (μm).

Specimen No.	1	2	3	4	5	6	7	8	9
Slurry thickness	26	38	42	59	60	76	81	98	107

**Table 3:** Coating surface stats parameters.

Surface Stats	#1	#2	#3	#4	#5	#6	#7	#8	#9
Ssk	0.182	−0.202	−0.0418	−0.00552	0.144	0.154	0.104	−0.0864	−0.390
Sku	3.08	3.35	4.15	4.23	3.59	3.66	4.06	4.40	3.12
Sv (μm)	26.1	29.6	54.6	36.8	27.0	23.2	31.0	55.8	74.3
Sq (μm)	39.5	25.4	53.3	43.2	36.4	36.4	37.7	83.2	70.8

## Data Availability

Not applicable.
